# Integrating high‐speed videos in capture‐mark‐recapture studies of insects

**DOI:** 10.1002/ece3.7372

**Published:** 2021-05-02

**Authors:** Rassim Khelifa, Hayat Mahdjoub, Leithen K. M’Gonigle, Claire Kremen

**Affiliations:** ^1^ Department of Zoology and Biodiversity Research Centre University of British Columbia Vancouver BC Canada; ^2^ Department of Biological Sciences Simon Fraser University Burnaby BC Canada; ^3^ Institute for Resources, Environment and Sustainability University of British Columbia Vancouver BC Canada

**Keywords:** capture–mark–recapture, demography, ecology, insects, recapture, survival

## Abstract

Capture–mark–recapture (CMR) studies have been used extensively in ecology and evolution. While it is feasible to apply CMR in some animals, it is considerably more challenging in small fast‐moving species such as insects. In these groups, low recapture rates can bias estimates of demographic parameters, thereby handicapping effective analysis and management of wild populations. Here, we use high‐speed videos (HSV) to capture two large dragonfly species, *Anax junius* and *Rhionaeschna multicolor*, that rarely land and, thus, are particularly challenging for CMR studies. We test whether HSV, compared to conventional “eye” observations, increases the “resighting” rates and, consequently, improves estimates of both survival rates and the effects of demographic covariates on survival. We show that the use of HSV increases the number of resights by 64% in *A. junius* and 48% in *R. multicolor*. HSV improved our estimates of resighting and survival probability which were either under‐ or overestimated with the conventional observations. Including HSV improved credible intervals for resighting rate and survival probability by 190% and 130% in *A. junius* and *R. multicolor*, respectively. Hence, it has the potential to open the door to a wide range of research possibilities on species that are traditionally difficult to monitor with distance sampling, including within insects and birds.

## Graphical abstract

Capture–mark–recapture (CMR) is a very useful technique to estimate demographic parameters.Many highly mobile animals are challenging to recapture after release, but high‐speed videos (HSV) could capture the mark on the individuals.We show that integrating HSV increases the recapture rate and improves estimates of survival and the effect sizes of important covariates.

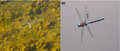

## 1 INTRODUCTION

Capture–mark–recapture (CMR) is a widely used sampling and statistical method in animal ecology, evolution, and biological conservation (Amstrup et al., 2010; Lettink & Armstrong, 2003; McCrea & Morgan, 2014; Pradel, 1996; Williams et al., 2002). It enables inferences about population size, survival rates, recruitment, and dispersal, as well as inferences about how both intrinsic (e.g., body size, age, disease) and extrinsic factors (e.g., temperature, precipitation, food) (Conn & Cooch, 2009; Hassall et al., 2015; Martins et al., 2011; Rose et al., 2018) affect these demographic measures. Depending on the taxa, capturing and marking animals in the wild can be a difficult task and stressful experience for the handled individuals. Recapturing marked individuals is also a challenging process, even in systems where individuals do not disperse. These difficulties are enhanced for highly mobile animals such as mammals, birds, and insects (Dickinson et al., 1999; Muijres et al., 2012).

Insect species can be good model organisms for CMR studies (Hagler & Jackson, 2001; Mollet et al., 2015; Schtickzelle et al., 2003). Besides their smaller size which allows convenient capture, handling, and marking, they also have a short life cycle, conspicuous behavior, small home‐range size, and fixed adult size. Identifying a marked individual often requires immobilizing it, as small size and rapid speed means that marks are typically difficult to detect while an insect is in motion. While repeated physical captures enable identification of marked individuals (Hagler & Jackson, 2001), repeat captures of insects may alter their behavior and/or cause injuries, thereby affecting their survival (Cordero et al., 2002; Morton, 1982). Telemetry is another possible solution, but it is still technologically challenging and costly to miniaturize transmitters to a size that suitable for most insects (Kissling et al., 2014). Consequently, most CMR studies have been carried out with conventional direct observations of marks on insects, but these often yield low recapture rates and, subsequently, produce unreliable demographic and/or dispersal estimates (Service, 1993). Such poor estimates can hinder conservation planning (Hammill & Clements, 2020). To obtain higher recapture rates, it is necessary to increase the amount of sampling efforts which often requires a larger number of surveyors, increased financial cost, and greater ecological disturbance.

One of the best represented insect groups in CMR studies is odonates (dragonflies and damselflies) (Conrad et al., 1999; Cordero‐Rivera & Stoks, 2008; Kéry & Juillerat, 2004; Khelifa et al., 2016). Usually, for species that regularly perch and do not consistently move, an alphanumeric code is written on the wing with a permanent marker. For active species that often fly, an alternative marking method is to use colored spots on the wings to identify flying dragonflies without capture (Cordero et al., 1999; Jacobs, 1955). In all cases, odonates can be particularly challenging to resight after release, particularly in the large species (especially those in the Aeshnidae and Libellulidae families) because they rarely perch and spend most of their time in flight where they are too fast for the human eye to read any mark (Corbet, 1999). With such conventional visual observations only, resighting rates are typically low. To overcome this, one potential tool that does not require the physical capture of individuals is high‐speed video (HSV), where video footage is recorded with a high enough frame rate that, when slowed down, enables identification of an individual via markings on their wings (Li et al., 2018).

HSV has been used in the study of animal behavior, particularly during the last few decades. Whether it is flying, running, swimming, feeding, or drinking, high‐speed videos have enabled scientists to examine diverse behaviors of various taxa meticulously (Altshuler et al., 2004; Maie et al., 2009; Marras et al., 2015; Reis et al., 2010). The rapid recent uptake of this technology in ecology is due to the substantial decrease in the cost of such cameras, along with reductions in camera size and weight and improvements in video duration and quality (Garbin, 2018; Lailvaux, 2018; Steen, 2014). Although HSVs have been used to study various aspects of animal behavior, the utility of HSV in CMR studies of highly mobile animals has not been tested.

Here, we assess how HSV can contribute to the resighting of marked individuals and ask whether such a method can improve estimates of demographic parameters. Using populations at artificial ponds, we carried out CMR on two large North American dragonflies, *Anax junius* and *Rhionaeschna multicolor*, and then used the Cormack–Jolly–Seber model to compare the resighting and survival rate of the capture history generated by the conventional visual method (CV: direct observation with the naked eye) to that generated by combining CV with high‐speed video (CV + HSV). Further, we tested whether HSV improves our ability to estimate the effect sizes of covariates that might impact survival, such as body size and age.

## 2 MATERIAL AND METHODS

### 2.1 Study site

The study was conducted in the experimental ponds of the University of British Columbia, Vancouver, Canada (49°14'56" N, 123°14'02" W). The site has a total of 20 artificial ponds, each of dimensions 15 × 25 m^2^, arrayed across an area of 130 × 100 m^2^. The two dominant species we studied, *Anax junius* and *Rhionaeschna multicolor,* are among the largest and highly mobile dragonflies in North America (Paulson, 2009).

### 2.2 Field sampling

**FIGURE 1:**
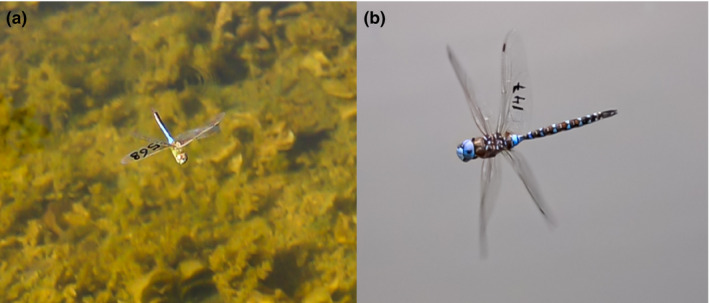
Still images taken from high‐speed videos showing the mark on the hind wing of the two studied dragonflies. (a) *Anax junius* and (b) *Rhionaeshna multicolor*. These marks on the wing are typically not detectable to the naked human eye when these dragonflies are in flight

We carried out daily CMR between 6th June and 24th July 2020 from 11:00 to 17:00 by capturing mature individuals with hand nets and marking them with a permanent marker on the hind wing. No visit was carried out on days with bad weather, because dragonflies are not active (number of days = 12). Two observers walked across the study site to capture unmarked individuals and tag recovery. Individuals were mostly captured on flight, but also in copula or oviposition. Individuals were immobilized by holding their wings, which reduces the disk of wing damage during marking and measuring. To release marked individuals, we gently placed them on a plant support and they typically took off shortly after. To obtain an estimate of body size, hind wing length was measured with an electronic caliper to the nearest 0.01 mm. The detection of whether an individual was marked or not was easy, but identifying the code on flight (the most common activity) was often not possible. We used two resighting methods: (1) conventional observations with the naked eyes (CV), and (2) high‐speed videos using the Sony RX10 Mark4 (cost:~1,700 USD) with 250 fps high‐frame‐rate mode, which allows slowing the motion by 8–10 times. The HSV camera does not require the user to have an eye on the viewfinder. The videos were later visualized for identifying the individual code (Figure 1). We asked how the use of a high‐speed camera increases the number of resightings relative to conventional observations alone, and, further, whether inclusion of camera data can improve estimates of survival and accompanying covariates (age and body size).

### 2.3 Important sampling notes

It is important to point out that most CV observations were made when the species were perched (rare behavior in reproductive sites), in copulation or oviposition, thus detectable with the naked eye. In these cases, it was not necessary to use HSV as the individual was already recorded with CV. HSV was typically used when individuals were in flight and nearly impossible to identify with the naked eye. Thus, observations acquired with CV and HSV cover different behaviors and rarely overlap. If an individual was observed with both CV and HSV on the same day, the HSV observation was discarded since it was redundant. Instead of comparing CV with HSV, we are interested here in assessing how HSV can *supplement* data that could not be observed with CV and how this potential increase in the resolution of data might improve demographic estimates. To accomplish this goal, we compare CV with CV + HSV.

### 2.4 CMR Data

For each species, we constructed two types of binary capture histories for each individual. The first involved only conventional observations, collected with the naked eye (CV dataset), whereas the second included both the conventional and high‐speed video observations (CV + HSV dataset). Both datasets involved 39 time occasions (days) and the same number of marked individuals: a total of 107 *A. junius* (74 males and 33 females) and 152 *R. multicolor* (96 males and 56 females).

### 2.5 Cormack–Jolly–Seber model (CJS)

CJS model is a powerful tool to estimate the survival rate of marked individuals using live encounter data (Lebreton et al., 1992). The model accounts for imperfect detection, gives estimates of the survival and resighting probabilities, and tests how the latter are determined by intrinsic factors such as age, body size, and extrinsic factors such as the weather (Kéry & Schaub, 2012). In odonates, many studies have used CJS to estimate rates of recapture and survival (Cordero–Rivera et al., 2019; Khelifa et al., 2019; Macagno et al., 2008; Minot et al., 2020; Outomuro et al., 2016: Stoks, 2001b).

We implemented the CJS model using the individual state‐space formulation as described by Gimenez et al., (2007). In a survey involving *n* individuals and *T* encounter occasions, we first define the observations, then the states of the individual. We define *X_i_*_,_ *_t_* as the binary random variable which is equal to 0 if the individual *i* is dead at time *t* and equal to 1 if the individual *i* is alive at time *t*. We then define *Y_i_*_,_ *_t_* as the binary random variable which is equal to 0 if the individual *i* is not encountered at time *t* and equal to 1 if the individual *i* is encountered at time *t*. The parameters involved in the likelihood are Φ*_i,t_* which is the probability that the individual *i* survives to time occasion *t* + 1 given that it is alive at time occasion *t* (*t* = 1, 2, …, *T*‐1), and *p_i,t_* which is the probability of recapturing (resighting) individual *i* at time occasion *t* (*t* = 2, …, *T*). The initial occasion where individual *i* is observed is denoted *e_i_*. The general state‐space formulation of the CJS model includes the observation Equation (1) and the state Equation (2) where *t* ≥ *e_i_* and $p_{i,e_{i}}=1$.

(1) $$Y_{i,t}|X_{i,t}∼\text{Bernoulli}\left(X_{i,t}p_{i,t}\right)$$

(2) $$X_{i,t+1}|X_{i,t}∼\text{Bernoulli}\left(X_{i,t}\Phi_{i,t}\right)$$

This approach disentangles the actual demographic process from its observation in the field. The model works such that when individual *i* is alive at time *t*, it has probability *p_i,t_* of being recorded and probability 1−*p_i,t_* of not being recorded, which translates into *Y_i,t,_* which follows a Bernoulli distribution with success parameter equal to the product of *p_i,t_* and *X_i,t_*. If the individual is not alive (*X_i_*_,_ *_t_* = 0), it cannot be detected because *X_i,t_* × (*p_i,t_*) = 0, and if it is alive (*X_i_*_,_ *_t_* = 1), it is detected with probability *X_i,t_* × (*p_i,t_*) = 1 × (*p_i,t_*) = *p_i,t_*. The implementation of the model was carried out with JAGS using MCMC technique (Brooks et al., 2000; Gimenez et al., 2009).

All statistical analyses were carried out using R 3.4.0 (R Development Core Team, 2020). We implemented the MCMC algorithm using JAGS software (Plummer, 2003) within R. Specifically, we used the following packages: rjags (Plummer, 2019), R2jags (Su & Yajima, 2020), and runjags (Denwood, 2016). We specified noninformative prior distributions for all parameters. Specifically, we used flat normal distributions for priors (dnorm(mean = 0, precision = 0.01)) for both survival and resighting probabilities, as well as the intercept and slopes of covariates (age and wing length). We tested for the effect of sex on resighting probability and effects of age and wing length on survival probability. We ran three independent chains, each for 10,000 iterations, omitting the first 1,000 as burn‐in and thinning every 100 iteration to reduce autocorrelation. We report posterior means as point estimates and Bayesian credible intervals. The code of the models is posted in the supplementary material.

## 3 RESULTS

### 3.1 Number of resightings

With CV observations, we resighted 28 (26.1%) marked *A. junius* and 25 (16.4%) marked *R. multicolor* at least once. With CV + HSV observations, we resighted 46 (43%) *A. junius* and 37 (24.3%) *R. multicolor* individuals, which correspond to proportional increases in recaptures of 64.2% and 48%, respectively.

### 3.2 Resighting probability

**FIGURE 2:**
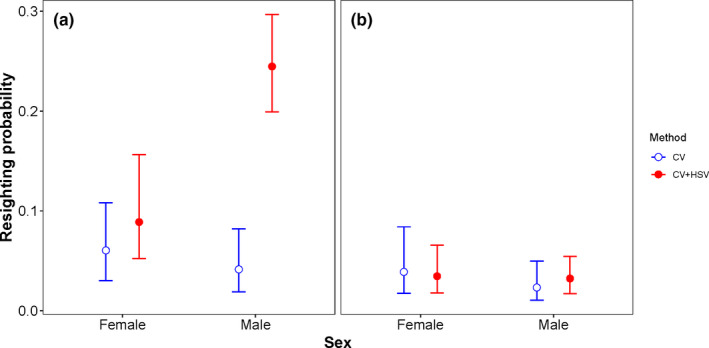
Resighting probabilities of females and males of the two dragonfly species monitored during capture–mark–recapture using conventional observations (CV) and integrating high‐speed videos (CV + HSV). (a) *Anax junius*. (b) *Rhinoaeschna multicolor*. Error bars are the 95% credible intervals

The average resighting probability estimated with CJS model was higher with CV + HSV than with CV in both species. In CV and CV + HSV, respectively, resighting probability was 0.05 ± 0.01 and 0.19 ± 0.02 in *A. junius* and 0.05 ± 0.01 and 0.06 ± 0.01 in *R. multicolor*. The effect of sex on resighting probability was revealed with CV + HSV but not with CV in *A. junius*, showing higher resighting probabilities for males than females (Figure 2a). This sexual difference was not observed in *R. multicolor* (Figure 2b).

### 3.3 Survival probability

We estimated a slight difference in the average survival rate when using CV versus CV + HSV in both species. In *A. junius*, using CV and CV + HSV, respectively, we estimated average survival probability to be 0.90 ± 0.02 and 0.87 ± 0.01, and in *R. multicolor*, we estimated average survival probability to be 0.84 ± 0.02 and 0.88 ± 0.03. Table 1 presents the survival estimates of males and females using CV and CV + HSV in both species.

**TABLE 1:** Survival estimates of female and male *Anax junius* and *Rhionaeschna multicolor.* Estimates were derived from a Bayesian Cormack–Jolly–Seber model

Species	Sex	Method	Mean survival	LCI	UCI	Rhat
*Anax junius*	Female	HFR	0.928	0.849	0.977	1.009
*Anax junius*	Female	No HFR	0.986	0.860	0.99	1.004
*Anax junius*	Male	HFR	0.883	0.844	0.917	1.000
*Anax junius*	Male	No HFR	0.931	0.816	0.999	1.005
*Rhionaeschna multicolor*	Female	HFR	0.831	0.731	0.913	1.002
*Rhionaeschna multicolor*	Female	No HFR	0.845	0.733	0.932	1.001
*Rhionaeschna multicolor*	Male	HFR	0.975	0.866	1.00	1.023
*Rhionaeschna multicolor*	Male	No HFR	0.907	0.750	0.998	1.036

**FIGURE 3:**
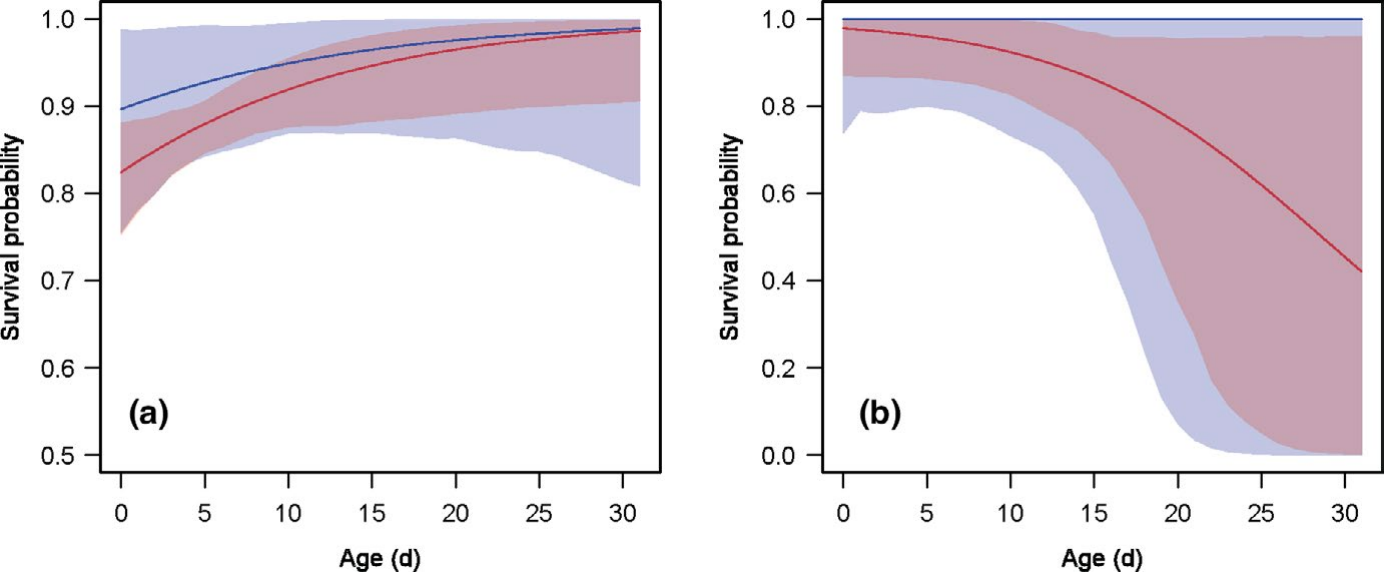
The effect of age on the survival probability of the two dragonfly species monitored during capture–mark–recapture using conventional observations (CV: blue) and integrating high‐speed videos (CV + HSV: red). (a) *Anax junius*. (b) *Rhinoaeschna multicolor*. Error bars are the 95% credible intervals

**FIGURE 4:**
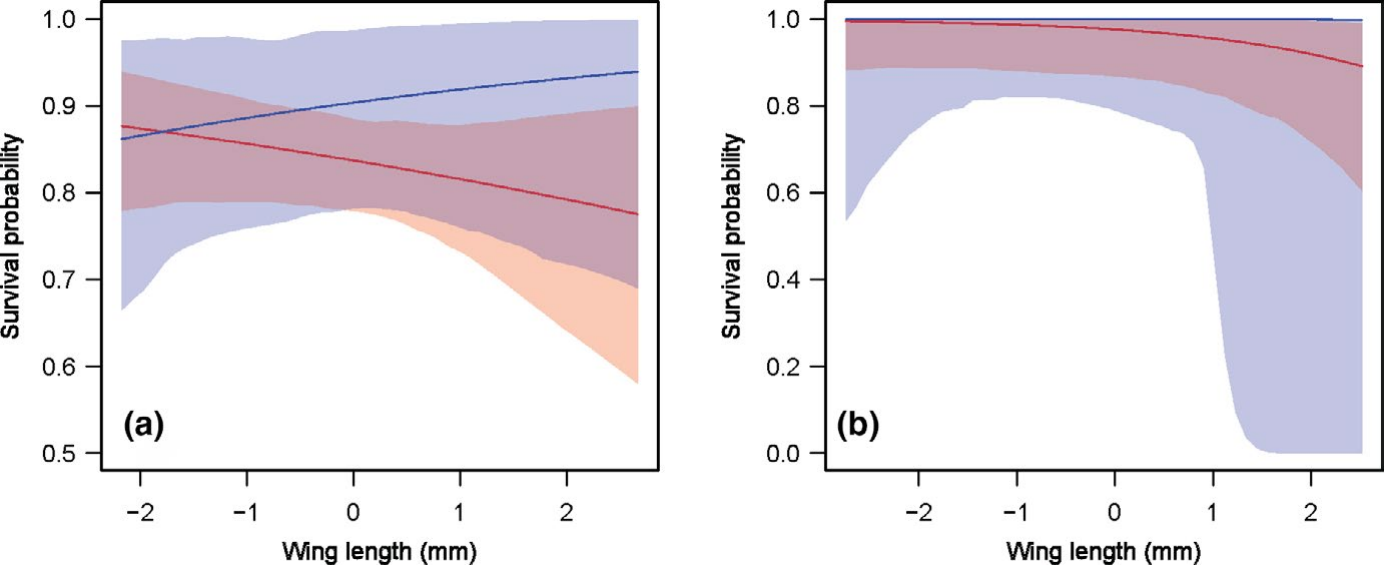
The effect of wing length on the survival probability of the two dragonfly species monitored during capture–mark–recapture using conventional observations (CV: blue) and integrating high‐speed videos (CV + HSV: red). (a) *Anax junius*. (b) *Rhinoaeschna multicolor*. Error bars are the 95% credible intervals

The two methods showed differences in the pattern of survival probability across age and wing length. Both methods showed a slight increase in survival probability with age in *A. junius*, although the mean was slightly overestimated using CV (Figure 3a). However, different patterns were found in *R. multicolor* where CV showed a constant survival probability across age whereas CV + HSV showed a negative effect of age (Figure 3b). In *A. junius*, CV showed a positive effect of wing length on survival probability whereas CV + HSV showed the opposite pattern (Figure 4a). No significant effect of wing length on survival was detected for *R. multicolor* using both methods (Figure 4b).

### 3.4 Credible intervals

In both species, CV + HSV produced estimates with smaller credible intervals for survival probability (Figures 3, 4). The credible intervals were 1.9 and 1.3 times smaller for the average resighting probability, and 2 and 1.3 times smaller for the average survival probability in *A. junius* and *R. multicolor*, respectively. Similar reduction of the credible intervals was observed in the analysis of the effect of age (Figure 3) and wing length (Figure 4) on survival probability.

## 4 DISCUSSION

Our study demonstrates that the use of a high‐speed camera increases the number of resightings and improves certainty of estimates of demographic parameters. Our analyses showed that HSV provided additional data that increased the number of resightings in our CMR data, improved estimates of resighting and survival probabilities, and reduced the uncertainty around the effect sizes of important covariates such as age and body size. Such improvements can make a big difference in the monitoring of survival rates, recruitment, or population size of wild populations. These findings highlight the importance of integrating HSV in CMR studies as an easy noninvasive tool to track wild populations of various animals including those of conservation concern. Such integration could potentially enable new applications of findings from population dynamic surveys of insects, as well as allowing for the potential study of fast and highly mobile animals.

### 4.1 Implications of improving resighting estimates

Understanding the reason for the nondetection of species is crucial to make behavioral and ecological inferences. In our study, HSV increased the resighting rate substantially in *A. junius* but not *R. multicolor*. This means that in the former species the marked individuals were present at the site but the observers were not able to detect them with the naked eye (false negative), whereas in the latter species the low resighting rate under both CV and CV + HSV was likely due to the absence of the individuals at the site. This interspecific difference in resighting rate is probably due to the difference in adult behavior where *R. multicolor* might be more dispersive than *A. junius* (Conrad et al., 1999). Furthermore, our analysis of *A. junius* capture history showed that HSV might reveal sex‐specific differences in resighting rates that could not be detected by CV. This discrepancy may lead to erroneous inferences about species behavior and the dismissal of sexual differences in species movement and habitat use (Fujiwara et al., 2006; Stoks, 2001a). Moreover, exhaustive estimates of resighting rates may provide more power to detect traits that drive reproductive success, in particular, and evolution, in general. In many insects, single individuals rarely land in breeding sites, whereas breeding pairs perch to copulate and lay eggs. This behavioral difference between breeding and nonbreeding individuals poses a challenge for researchers interested in characterizing the fitness landscape and identifying how trait values correlate with fitness. Also, since resighting rate is an important component in demographic models, enhancing the estimation of resighting rates may lead to a more accurate evaluation of population size (Tufto et al., 2012), and ultimately to more effective management of wild populations (Hammill & Clements, 2020). Therefore, the improvement of our estimates of resighting rates by HSV might resolve many issues when addressing ecological, conservation, and evolutionary questions.

### 4.2 Implications of improving survival estimates

Data on survival probability are crucial for demographic modeling (Carey, 1993). The estimated survival probability in *A. junius* was similar to that reported for the congeneric A. *imperator* surveyed with conventional capture–mark–recapture in Northwestern France (Minot et al., 2020). Our results show that HSV improved the estimates of survival probability in both species. Such an improvement might increase the reliability of our predictions of population trends (Carey, 2001). Many scientists are interested in the effect of age on survival to investigate evolutionary theories related to senescence (Bonduriansky & Brassil, 2002; Sherratt et al., 2010, 2011; Zajitschek et al., 2020). While this is theoretically feasible with CMR of wild population of insects, field studies based on the naked eye yield low‐resolution data (low resighting rates) which prevents accurate predictions. In our study, we showed that HSV reduced the uncertainty around survival estimates across age and made the age‐dependent survival pattern more detectable. Similarly, HSV improved our estimates of body size effects on survival probability. This is particularly important because body size has been the focus of many studies in evolutionary biology and ecology (Blanckenhorn, 2000; LaBarbera, 1989; Ozgul et al., 2014).

## 5 CONCLUSION

We believe that HSV could be a valuable noninvasive, economic, and practical tool in CMR. The advantages of using HSV include improving data quality, minimizing the physical damage to the study species, reducing sampling efforts, and saving money. Another advantage of HSV is that it can capture image of multiple marked flying individuals at the same time, whereas human vision can focus only on a single target. The integration of HSV in CMR is timely because there have been remarkable technological advances in high‐speed video capabilities during the past decade, with new cameras now able to capture high‐quality HSV, longer videos, lenses with better telephoto range, and processors that can handle recordings rapidly. Digital cameras have high‐frame‐rate video modes at reasonably low costs (Steen, 2014). HSV could also be used in CMR of highly mobile birds and mammals. Future methodological integration of high‐speed camera might be in camera‐trapping‐based studies (Rico‐Guevara & Mickley, 2017) to investigate demographic parameters, dispersal, home range, and behavior.

## CONFLICT OF INTERESTS

The authors have no competing interests, financial or otherwise, to declare.

## AUTHOR CONTRIBUTIONS

RK and HM did the fieldwork. RK and LM analyzed the data. RK conceived the idea and led the writing of the paper with contribution from all authors who gave final approval for publication.

## Supporting information

Video S1

## Data Availability

The full dataset and R code are deposited in Dryad repository: https://doi.org/10.5061/dryad.t76hdr80f
